# Follicular Metabolites-Assisted Clinical Evaluation of IVF/ICSI Outcomes

**DOI:** 10.1155/2021/9999659

**Published:** 2021-05-26

**Authors:** Bing Qu, Yunhe Xiong, Xiaofan Yu, Jinli Ding, Jing Weng, Xinghua Yang, Yanmin Ma, Lingyan Liu, Jing Yang

**Affiliations:** ^1^Reproductive Medicine Center, Renmin Hospital of Wuhan University, Wuhan, Hubei Province, China; ^2^Urology Department, Renmin Hospital of Wuhan University, Wuhan, Hubei Province, China; ^3^School of Pharmaceutical Sciences, Capital Medical University, Beijing, China; ^4^School of Basic Medical Sciences, Capital Medical University, Beijing, China; ^5^School of Public Health, Capital Medical University, Beijing, China; ^6^Beijing Obsterics and Gynecology Hospital, Capital Medical University, Beijing, China

## Abstract

As infertility became a significant public health problem, assisted reproductive technologies (ARTs) were introduced. However, the fertilization rate of in vitro fertilization (IVF) per cycle varied, and patients needed to repeat IVF or change to intracytoplasmic sperm injection (ICSI). Here, 75 couples suffering from female fallopian tubal blockage (tubal group) and 42 spouses beset by male abnormal sperm status (dysspermia group) were recruited. We comprehensively explored the relationship among couples' clinical factors, follicular metabolites, and IVF/ICSI stepwise outcomes. IVF/ICSI outcomes were affected by follicular metabolites and physical status in both women and men, regardless of which side infertility came from. Particularly, in the tubal group, the energy supporting pathways—glycolysis and pyruvate metabolism—were most essential in follicles, and IVF/ICSI outcomes were also related to sperm parameters. However, in the dysspermia group, in addition to sperm conditions, oocyte quality acted as a compensation for poor sperm quality, for which aminoacyl-tRNA biosynthesis and the related supporting metabolism were critical in the follicular environment, and ultimately played a decisive role in IVF/ICSI outcomes. The respective logistic regression models in combination with selective male sperm parameters, estradiol (E2), follicular alanine, glutamine, glycoprotein, lipid, and acetic acid, were constructed to predict IVF or ICSI outcomes. No matter which sex infertility comes from, factors from both men and women should be considered. The current study provides a feasible option for pre-IVF evaluation, as well as guidance for follow-up clinical intervention to improve IVF/ICSI success rates.

## 1. Introduction

Data from both the United States [1] and UK [2] governments pointed out that 12–15% of couples suffered infertility in their productive age, which was identified as a significant public health issue. The most common cause of female infertility was fallopian tube problems, affecting over 25% of infertile women. The structural abnormality and/or pelvic inflammation stops spermatozoa from traveling in the fallopian tube and subsequently fertilizing locates [3]. About half of the infertilities involve male factors, either the male factor alone or male-female combined problems, while most of the male infertility is preventable, ameliorable, or treatable [4]. Invasive tools were introduced to assist infertile couples. However, the fertilization rate per cycle varied widely based on assorted issues including disease, ovulation, induction control, oocyte selection, operator's experience, and ART processes. A large proportion of patients need to repeat IVF or switch to ICSI for satisfactory fertilization. This not only brings physical but also financial burdens to patients.

Efforts have been carried out toward improving IVF outcomes. Summarizing overall biological events, metabolites can reflect system dynamics into a series of detectable small molecules with sophisticated modern metabolomic techniques [5]. Hence, besides blood cytology or biochemical indicators, some researchers targeted metabolites from the embryo culture medium (ECM) [6, 7], follicular fluid [8, 9], and female blood [10] for biomarkers to predict IVF outcomes. However, inconsistent findings exist in different studies [2]. It is presumably because metabolic pictures in the productive area were far more complex than those diseases whose status can be considered in single organs. The combination of sperm and oocytes, as well as the following zygotic development, involved both paternal and maternal individuals. Hence, both females' and males' physical and endocrine status, in addition to follicular metabolic profiling, could have nonnegligible impacts on reproductive outcomes.

In the oocyte environment, the follicular fluid includes information on granulosa cell secretion and conduction, as well as metabolites released during oocyte growth [11]. In our previous study of altered follicular metabolic profiling in PCOS patients, we discovered significant effects of common clinical characteristics on follicular metabolites, which potentially influence IVF outcomes [9]. To expand on it, the present study aims to investigate both male and female clinical factors, as well as follicular components that influence IVF outcomes in two different disease groups, infertility with male sperm problems and infertility with female fallopian tube problems. After collecting the follicular metabolite information by ^1^H NMR, we firstly identified the influence of females' physical and endocrine parameters on follicular metabolites. With this knowledge, we observed the relationships of IVF/ICSI outcomes with couples' clinical parameters and follicular metabolites in two different disease groups. Logistic regression models were constructed to predict IVF results, to assist clinicians to evaluate the success rates of IVF, and to determine whether to conduct IVF or ICSI directly.

## 2. Experimental Section

### 2.1. Sample Collection

This study involved 117 couples who underwent IVF/ICSI in Renmin Hospital of Wuhan University, China. Among them, 75 couples suffered from female fallopian tubal blockage (tubal group) without any male problems. The other 42 couples were purely affected by male abnormal sperm status (dysspermia group), consisting of 27 oligozoospermia and 15 azoospermia cases. Sperm status was measured according to WHO-5 standards [12]. No other reproductive or endocrine diseases were found in this sample population. The whole sample cohort included Chinese females from Hubei, China. The study was approved by the Institutional Review Board (IRB) of Renmin Hospital of Wuhan University, China. Written consent has been obtained from each patient or subject after a full explanation of the purpose and nature of all procedures used.

All recruited females were advised to have proper meals for at least seven days before ovulation. Oocytes were stimulated by the GnRH-long protocol. Sex hormone levels were measured, and ultrasonography was performed regularly. Follicles were collected 34–36 hours after injection of human chorionic gonadotropin (HCG). Oocyte retrievals were carried out under the guidance of transvaginal ultrasound. The follicular fluid from the same patient was combined and centrifuged at 12000 rpm for 10 min under 4°C to remove tissue, debris, and granulosa cells. Finally, the supernatant was transported under dry ice and stored at −80°C until analysis. The relative clinical information of the couples was collected.

### 2.2. Assisted Reproductive Technology (ART) Protocol and Information

This study included only one cycle per woman. After oocyte retrieval, insemination underwent IVF within three hours using gradient-prepared spermatozoa in the fertilization medium. The appearance of two pronuclei (2PN) was observed closely, and intracytoplasmic sperm injection (ICSI) was performed also within 4 hours if necessary. Thirty couples received ICSI directly because they had at least one of the following problems, (1) sperm activity rate (level a + b) < 1%; (2) sperm density < 1×10^6^/ml; (3) normal sperm morphology rate <1%; (4) fertilization failure in IVF in the last ART cycle. The oocytes were then transferred to a cleavage medium for further development. The overall fertilization rate (FR) was calculated as the ratio of successfully fertilized oocyte number to the oocyte number that was involved in the insemination. Next, the number of 2PN was counted under a microscope. The ratio of the 2PN number to the fertilized oocyte number was assigned as the effective fertilization rate (2PN-FR). The standard embryo quality evaluation strategy was exerted three days after cleavage [13] as in Table S1. Those embryos belonging to grade I or II and blastomeres numbering between six and eight on day three were defined with top quality. The top-quality embryo rate (TQER) was calculated as the ratio of the top-quality embryo number to the 2PN cleavage number.

### 2.3. ^1^H NMR Experiments

Frozen follicular fluid samples were thawed, vortexed, and centrifuged. A volume of 530 *μ*L of each sample was transferred to a 5 mm NMR tube with a coaxial capillary containing 60 *μ*L TSP (0.53 mmol) in D_2_O, serving as the chemical shift and quantitative reference. The experiment sequence was randomized and carried out at 25°C on a Bruker DRX500-MHz NMR spectrometer using pulse sequences of CPMG (Carr-Purcell-Meiboom-Gill) with a water presaturation pulse. For each spectrum, 64 transients were collected and 16K data points were acquired using a spectral width of 6000 Hz. An exponential weighting function corresponding to 0.5 Hz line broadening was applied to the free-induced decay followed by Fourier transformation. Phasing and baseline correction was applied in Bruker XwinNMR software version 3.5.

### 2.4. Data Analysis

All statistical analysis was performed using SPSS Statistics (ver.21). For clinical parameters and IVF/ICSI outcomes, the average and standard deviation (Ave. ± STD) were provided, and a 95% confidence interval (CI) was used as the statistical threshold. A *T*-test, Mann–Whitney *U* test (for abnormally distributed data), and Chi-square test were used for comparison among groups. Pearson correlation was used to explore the relationship between variables. Since the IVF/ICSI results of FR, 2PN-FR, and TQER came from the step-by-step processes, a nonnegligible correlation existed among them. As a result, in the following sections, when observing the parameters that affected individual steps, the respective partial correlation coefficients were calculated with the control of the previous steps. In this way, the impacts from previous steps were removed. Moreover, for correlation calculation, a tightened significant threshold of *p* < 0.03 was chosen as the significant cutoff to minimize interference factors and false-positive results. Multivariate analysis was carried out by logistic regression analysis with a forward stepwise variable selection algorithm, which automatically retained the variables that are most conducive to discrimination. Nighty-five percent CI of model coefficients, as well as the prediction results of Receiver Operating Characteristic (ROC), were provided. Bonferroni adjustments of *p* values were performed using R software.

## 3. Results

### 3.1. Clinical Information

The sample cohort of this study involved 117 couples, including 75 couples in the tubal group and 42 couples in the dysspermia group. The clinical information for both female and male subjects was collected, and the representative demographic information including age, body mass index (BMI), luteinizing hormone (LH), follicle-stimulating hormone (FSH), and sperm density is summarized in Table 1 by group. Most of the characteristics were comparable between groups, except the sperm-related parameters (sperm density, sperm motility A and B, level A and level B sperm percentage, total motility, and PR + NP percentage), for which the tubal group was superior to the dysspermia group.

### 3.2. Follicular Metabolites Detected by ^1^HNMR

CPMG ^1^H NMR spectra focused on small molecules were collected for each follicular fluid sample. A total of 67 peak regions were identified with reference to their chemical shift and multiplicities [14]. Detailed information of these 67 peaks was listed in Table S2, including amino acids, glucose, choline, and lipids. The integrals of each peak region were obtained. Subsequent analysis with relevance to FF metabolites was employed on these 67 peak integrals.

### 3.3. Effects of Clinical Parameters on Follicular Metabolites

**Figure 1:**
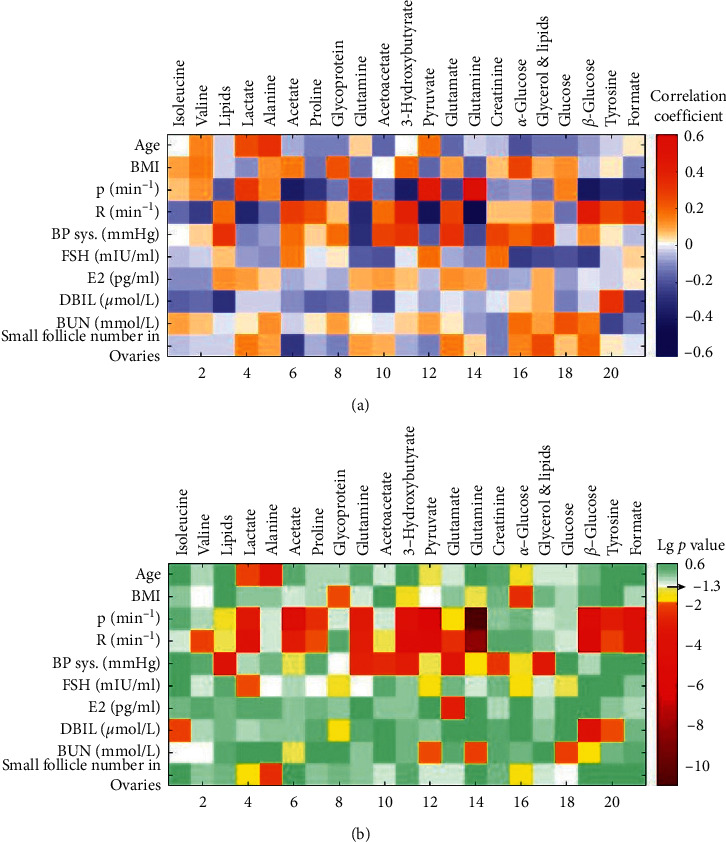
(a) Pearson's correlation coefficients between clinical parameters and NMR detected metabolites. The stained color represents correlation coefficients ranging from −0.6 to 0.6. (b) Pearson's correlation *p* values of each paired data. The visual color represents log 10 (*p* value) ranging from −11 to 0.6. The pairs with *p* value of 0.05 and 0.01 were dyed light yellow and orange, respectively.

Correlation analysis was performed on 67 follicular metabolite peak integrals of 117 female patients and their clinical parameters. The correlation coefficients and corresponding log 10 based *p* values are visually presented in Figure 1. Those pairs shown in orange to dark red colors were closely related pairs, having a correlation with a significant *p* value less than 0.01.

Firstly, the women's pulse rate (P. min^−1^), respiration rate (R. min^−1^), and systolic blood pressure were the most effective clinical parameters. P. and R. acted reversely on follicular metabolites, including lactate, acetate, proline, glutamine, 3-hydroxybutyric acid, pyruvate, glutamate, *β*-glucose, tyrosine, and formic acid. The influential trends of BP sys. on follicular metabolites were almost the same as R. Secondly, liver function index—DBIL—and renal function index—BUN—had an influence on isoleucine, *β*-glucose, tyrosine, and pyruvate, glutamine, and glucose. Thirdly, age performed a positive correlation with lactate and alanine levels. BMI was positively correlated with glycoprotein and glucose. Lastly, there is no doubt that endocrine parameters, E2 and FSH, affected the follicles. FSH had a negative impact on lactate, while E2 had a positive effect on glutamate. Besides, total small follicle number (SFN) in women's ovaries was positively associated with follicular lactate, alanine, and *α*-glucose (Figure 1).

### 3.4. IVF/ICSI Outcomes

**Figure 2:**
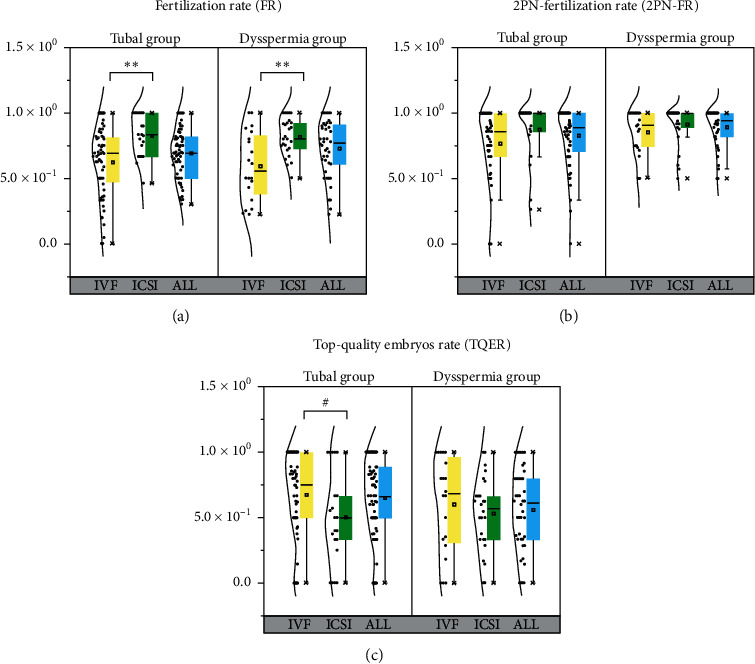
Group box plot for FR, 2PN-FR, and TQER.  ^*∗∗*^*p* value <0.01;  ^#^*p* value = 0.053.

In the whole sample cohort, 88 couples were treated by IVF, 47 couples underwent ICSI, and 18 spouses participated in both techniques. In Figure 2 and Table S3, the stepwise IVF/ICSI results in the forms of overall fertilization rate (FR), 2PN fertilization rate (2PNFR), and the top-quality embryo rate (TQER) were presented upon the technique used. The overall outcomes of both groups were summarized. There was no significant difference between the groups in each parallel comparison. For example, the IVF FR was similar in the tubal group (62.4 ± 26.9%) to that in the dysspermia group (59.3 ± 26.1%), and ICSI 2PNFR of the tubal group (87.3 ± 22.3%) was comparable with that of the dysspermia group (90.9 ± 15.1%c).

It was not surprising that, because of different insemination techniques, in both the tubal and dysspermia groups, the FR (around 80%) from ICSI treatments was clearly higher than that (∼60%) from IVF treatment (*p* < 0.01). Similarly, in the tubal group, 2PNFR of ICSI was elevated (*p* < 0.05). But, in the tubal group, TQER from IVF (67.6 ± 32.2%) exceeded the result from ICSI (50.2 ± 35.1%), with *p* < 0.05. When zoomed in the dysspermia group, this trend of higher embryo top-quality rate of IVF was also found in 15 couples with azoospermia, with the *p* value of 0.051 at the borderline.

### 3.5. Factors Affecting IVF/ICSI Outcomes in the Tubal Infertility Group

**Figure 3:**
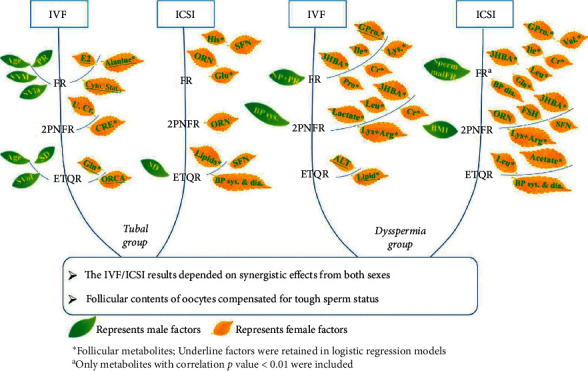
Summary of factors impacting stepwise IVF/ICSI outcomes by group. PR, progressive; NP, nonprogressive; Sperm NM, sperm normal morphology (%); Sperm Via., sperm viability; Sperm Vol., sperm volume; Sperm Den., sperm density; Sperm malFR, Sperm malformation rate; BP sys., systolic blood pressure; BP dia., diastolic blood pressure; FSH, follicle-stimulating hormone; E2, estradiol; Cyto. Stat., cytoplasmic states; U Cr., urinary creatinine; Gln, glutamine; Oocyte arr., oocyte radial crown arrangement (1. compact; 2. slightly dilated; 3. radial); SFN, small follicle number in ovaries; ORN, oocyte retrieved number; ALT, alanine aminotransferase; Ala, alanine; His, Histidine; Glu, glucose; GPro, glycoprotein; Lys, lysine; Ile, Isoleucine; Leu, leucine; Cr, creatinine; CRE, creatine; 3HBA, 3-hydroxybutyric acid; Val, valine; Gln, glutamine; Pro, proline.

Correlations between IVF/ICSI stepwise outcomes and couples' available clinical parameters were explored. Firstly, a significant positive correlation was found between FR and male age in both the IVF subset and the whole tubal group. It was not surprising that the IVF FR was positively correlated with sperm quality parameters, including normal sperm morphology and progressive motility (PR). But the ICSI FR was negatively correlated with oocyte retrieved number (ORN) and the total SFN from female ovaries (Figure 3, Table S4). Similarly, ORN appeared to have a negative effect on 2PNFR. According to the number of oocytes retrieved, we further divided the tubal ICSI cohort into two subgroups, one with the oocytes number greater than 15 (*n* = 9), and the other less than 15 (*n* = 12). The subgroup with more oocytes retrieved number had statistically lower FR (*p* < 0.05). Similarly, the 2PN rate was significantly reduced (*p* < 0.05) when the retrieved oocyte number achieved more than 15. The same negative trend was found in female urinary creatinine.

The embryo quality evaluation step was affected by more sophisticated factors. Again, sperm parameters and male age were influential. However, male age here negatively contributed to TQER, in comparison with its positive effect on FR. Like that in FR, the SFN in female ovaries was again negatively affecting ICSI EQTR. Also, female blood pressure was closely connected to ICSI TQER.

The follicular metabolites also presented a significant correlation with ART outcomes (Table S5). For patients who participated in ICSI, increased histidine and decreased glucose appeared to improve FR. Lactate and alanine seemed beneficial to overall ART FR. Creatinine played a role in the following 2PN transforming step, contributing positively to IVF 2PN-FR. Less glucose appeared profitable on overall ART 2PN-FR. Moreover, the high content of lipids presented helpful to ICSI TQER.

A summary of all factors impacting stepwise IVF/ICSI outcomes is presented in Figures 3 and S1 with more details.

### 3.6. Logistic Regression Predicting IVF/ICSI Outcomes for the Tubal Infertility Group

**Figure 4:**
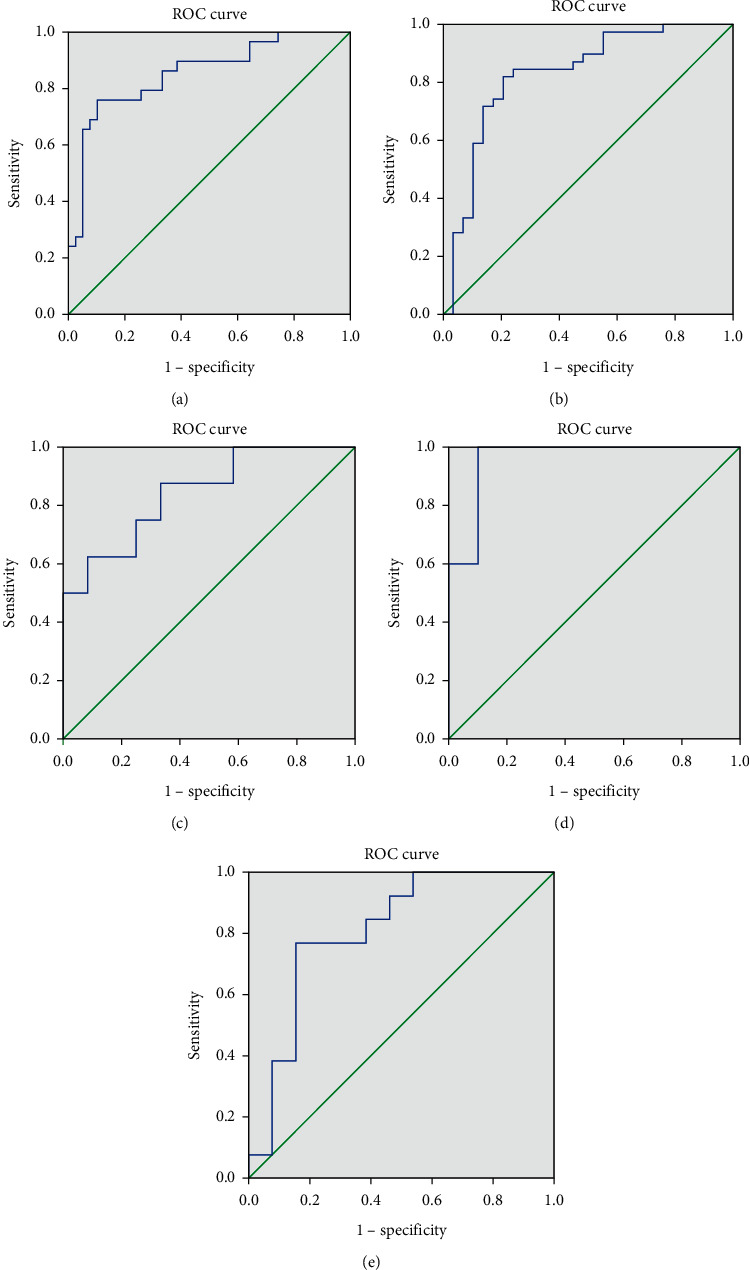
Plotted receiver operating characteristic (ROC) space from logistic regression prediction for (a) tubal group IVF FR, (b) tubal group IVF TQER, (c) dysspermia group IVF FR, (d) dysspermia group IVF TQER, and (e) dysspermia group ICSI TQER results.

In the tubal infertility group, patients who participated in IVF (*n* = 68) were further divided into two portions, one with the IVF FR less than 70% (*n* = 39, entered as “0”) and the other with IVF FR above 70% (*n* = 29; entered as “1”). The logistic regression model automatically retained the most discriminant variables with an overall predicted accuracy of 83.8%. ROC space is plotted in Figure 4, with the AUROC (area under ROC) of 0.85 (CI: 0.76∼0.95), illustrating a satisfactory discriminative ability. The coefficients of the included variables in the model, as well as their significance, 95% CI, are provided in Table 2. Higher levels of alanine and sperm viability, more homogeneous cytoplasm, and lower levels of E2 were favorable for IVF FR. Among them, follicular alanine was dedicated the most to the IVF FR with its high odds ratio (OR).

Similarly, tubal infertility couples that participated in IVF (*n* = 68) were split into two groups based on their TQER outcomes. Those with TQER lower than 70% were included in one group (*n* = 29, entered as “0”), and those with TQER higher than 70% were placed into another group (*n* = 39, entered as “1”). Follicular glutamine, arrangement of oocytes, radial crown, sperm volume, and viability were recruited in the final model generating 80.9% overall predicted accuracy. From the model parameters and sensitivity analysis, the glutamine level represented the greatest impact on IVF TQER. The model AUROC achieved 0.83 (95% CI: 0.73∼0.93, Figure 4). However, due to the limited ICSI sample population of this group, we were not able to construct any models for ICSI TQER.

### 3.7. Factors Affecting IVF/ICSI Outcomes for the Dysspermia Group

From the correlation results, it was not surprising to discover that sperm's overall progressive (PR) and nonprogressive (NP) motility composition was positively affecting IVF FR in the dysspermia group (Figure 3, Table S6). That is because, in IVF, sperm and oocytes are still self-combining, depending on the sperm's activity capacity. On the other hand, this activity was not as important as in the ICSI fertilization step. However, the harmfulness of sperm malformation on ICSI FR was highlighted. Moreover, women's endocrine and physical factors, including FSH, BMI, and systolic and diastolic blood pressure, also affected ICSI FR. A series of metabolites displayed influence on the fertilization step for dysspermia couples (Table S7). It demonstrated that glycoprotein, lysine, isoleucine, creatinine, and 3-hydroxybutyrate had had negative effects on FR in both IVF and ICSI methods. Proline affected IVF FR, while leucine, valine, and glutamine had an influence on ICSI FR.

In the dysspermia group, men's BMI and BP sys. exhibited importance in ICSI and IVF 2PN-FR, respectively. Women's FSH, ORN, and SFN displayed a negative correlation with 2PNFR again. During this step, 3-hydroxybutyrate, lysine, and arginine were negatively connected with both the IVF and ICSI results. The functions of creatinine, lactate, and leucine were more apparent with the IVF 2PN-FR outcome. In addition, males' blood pressure and follicular leucine were closely linked to the embryo's quality.

All factors impacting stepwise IVF/ICSI outcomes were summarized in Figures 3 and S1 with more details.

### 3.8. Logistic Regression Predicting IVF/ICSI Outcomes for the Dysspermia Group

In the dysspermia IVF group (*n* = 20), 12 couples obtained satisfactory IVF fertilization rates (FR ≥ 70%, *n* = 12, entered as “1”). The remaining couples received an FR of less than 70% (*n* = 8, entered as “0”). The forward stepwise algorithm only left the follicular glycoprotein in the logistic regression, but with 75% prediction accuracy and an AUROC of 0.84 (CI: 0.67∼1.00, Table 3). Half of these 20 couples received more than 70% IVF TQER (TQER≥70%, *n* = 10, entered as “1”). Both follicular lipids and female alanine aminotransferase (ALT) in blood appeared discriminative for IVF TQER, producing a high model prediction accuracy of 90.0% and an AUROC of 0.96 (CI: 0.87∼1.00, Figure 4). Among the retained variables, follicular lipids showed greater importance.

The patients who underwent ICSI were further divided into two halves, using a TQER of 60% as the cutoff. Logistic regression retained the only variable, follicular acetic acid, giving the model prediction accuracy of 76.9% and AUROC of 0.81 (CI: 0.64∼0.98, Figure 4).

### 3.9. Importance of Follicular Metabolism in IVF/ICSI Outcomes

**Figure 5:**
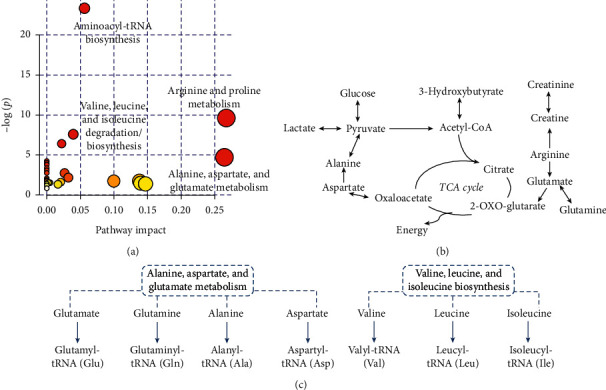
(a) Overview of fluctuated metabolisms in follicular fluid suggested by MetPA. (b) Metabolic pathway diagram showing perturbations in energy production metabolism involved essential follicular metabolites that were related to ART outcomes. (c) Metabolic pathway diagram showing perturbations in aminoacyl-tRNA biosynthesis involved follicular metabolites that were related to ART outcomes.

The metabolites that played roles in ART outcomes in the tubal and dysspermia groups were submitted to MetPA for pathway investigation. Based on their pathway impact and −log (*p*) from enrichment analysis, the important metabolisms involved in IVF/ICSI outcomes in the two groups were provided in Table S8 separately. The overall pathway map combining all related metabolites is presented in Figure 5. It mainly included aminoacyl-tRNA biosynthesis, arginine, and proline metabolism, as well as alanine, aspartate, and glutamate metabolism.

## 4. Discussion

In the present study, we focused on the two most common infertilities caused by the women's problem (fallopian tubal blockage) and the men's problem (sperm defects), respectively. We observed the influence of clinical indicators on follicular fluid metabolites in females and investigated the effects of follicular metabolites and couples' clinical parameters on IVF/ICSI stepwise outcomes. Our aim was to explore a feasible and objective method for IVF FR estimation and TQER evaluation, to assist the clinical staff to decide whether to carry out IVF or switch to ICSI directly. In the meantime, the comprehensive study revealed potential factors that influenced the IVF/ICSI outcomes, which could provide information for follow-up clinical intervention to improve the success rate.

From our data, the IVF TQER overwhelmed ICSI TQER, although ICSI had a much better FR due to its advanced insemination method. It suggested that although ICSI can improve fertilization, zygotic development depends more on the quality of both oocytes and sperm. In the tubal group (representing female infertility), the sperm's normal morphology and progressive motility exhibited importance only in the IVF fertilization process. Without the concern of sperm activity, the oocytes' parameter elevated more concerns in ICSI FR. However, sperm density was effective in the following TQER of both IVF and ICSI. In the dysspermia group (representing male infertility), both female physical factors and follicular metabolites had significant impacts on both FR and TQER, no matter which intervention approach was taken. It inferred that an index of follicular metabolites representing oocyte quality acted as a repairing role, compensating for the poor sperm quality, just like what it did in older male partners [15].

From our study, it is evident that clinical parameters, as well as their cross-influenced follicular metabolites, were closely associated with IVF/ICSI outcomes. Next, we will focus on these two aspects, respectively.

### 4.1. Clinical Parameters

BMI was positively associated with follicular glycoprotein in the current study, which was harmful in IVF fertilization of the dysspermia group as shown in the logistic regression model. Meanwhile, male BMI was also negatively associated with ART outcomes. Quite a few studies tried to correlate women's BMI with ART outcomes, but the results were conflicting because of reproductive intricacies [16]. Nevertheless, as the researchers pointed out, the BMI varied, and serum metabolites were reflected in follicular fluid, especially in protein concentration [17]. On the other hand, paternal obesity resulted in reduced reproductive potential, even with normality on conventional semen parameters [18], further supporting our findings.

Age is another concerning issue in the reproduction field. In our sample cohort, male age played a positive role in IVF FR, while it was harmful to high-quality embryo formation. A similar negative effect of male age was discovered in former studies where increased paternal age may induce adverse effects on sperm parameters [19], blastocyst embryo formation [20, 21], and IVF/ICSI success rate [19, 22]. The result could be caused by unexpected DNA fragmentation [23], chromosomal aneuploidies, and epigenetic mutation [22]. In females, age was negatively correlated with follicular glucose but positively associated with lactate, pyruvate, and alanine. This trend was also found in women with advanced age or impaired ovarian reserve, which accounts for the elevated glycolysis in granulosa cells [11]. In addition, we also observed that a negative correlation existed between female age and ORN. It suggested that the ovary stimulation protocol should be not only adjusted to the concern of disease but also according to female age and ovarian reserve capacity.

The ORN and SFN negatively correlated with not only ICSI and FR but also 2PNFR and TQER, respectively. The finding was coincident with a considerable retrospective study (*n* = 2 578) where transferable embryos declined with ORN [24]. In addition, from our results, as total SFN increased, several follicular metabolites altered, including lactate, alanine, and glucose, which were essential in IVF/ICSI outcomes. It suggested that the increased number of these two factors might result in decreased quality of oocytes. In both the tubal and dysspermia groups, smaller ORN and SFN were expected to achieve a more satisfactory outcome.

Female hormone levels impacted IVF/ICSI outcomes. In the tubal group, without other endocrine diseases, a lower E2 level would result in elevated FR, given by the regression model. Similarly, a lower E2 level was found in the higher clinical pregnancy rate group [25], and the higher E2 exposure group resulted in an increased rate of preterm delivery and a lower birth rate [26]. Besides, from the correlation analysis, increased FSH was favorable for both fertilization and 2PN FR. It was also illustrated that a higher FSH level within an appropriate range was plausible for good embryos' quality [27].

BP sys. and BP dia. represent the cardiovascular regulatory function. In both groups, negative correlations were found repeatedly between BPs and IVF/ICSI stepwise outcomes regardless of gender. On the other hand, we also observed that BP sys. had an impact on a series of follicular metabolites. It has been reported that men with higher BP had a lower quality of semen [28]. There is also great evidence supporting the association between high BP and declined renal function [29]. Some reports indicated that BP data was associated with end-organ health [30]. Furthermore, pulse rate and respiration rate, which reflect cardiac function, revealed their prominent role in multiple follicular metabolite fluctuations.

Female renal and liver function in terms of BUN and DBIL caused fluctuation of follicular composition. It was echoed by female urinary creatinine, another indicator of renal function, which possessed negative correlations with IVF 2PNFR in the tubal group. It was also consistent with follicular creatinine concentration, which correlated negatively with both FR and 2PNFR in the dysspermia group.

### 4.2. Follicular Metabolites

Follicular metabolite variation was attributed to the combinational effects of all physical factors. A series of metabolites eventually influenced IVF/ICSI outcomes, especially those showing discriminant properties in logistic regression models. In the tubal group, enriched follicular alanine and glutamine were favored for IVF, FR, and TQER, respectively. It is consistent with the finding that alanine supplementation was beneficial for embryonic developmental competence through increasing mRNA expression in pig oocytes [31]. It has been observed that follicular glutamine levels were positively correlated with FR in PCOS patients [32]. In addition, the lack of follicular glutamine resulted in low reproductive performance sows [33]. In the dysspermia group, glycoprotein was reserved as the only predictor in the IVF FR regression model, with a large negative coefficient. The increased glycoprotein could be an indicator of various inflammatory disorders, such as PCOS [32, 34], diabetes, cardiovascular disease, and infection [35]. For IVF TQER, elevated follicular lipid was expected. Although, in the NMR technique, we could not specifically identify the exact lipid, our previous MS data suggested that most of the glycerophospholipids and sphingolipids were lower in PCOS and were expected to increase for better IVF/ICSI outcomes [9]. A positive coefficient of acetic acid in the ICSI TQER model implied the demand of the acetyl group, which could provide the center for carbohydrates and fat metabolism when binding to coenzyme A. The usage of acetic acid could also perform as an alternative energy path just like in T cells [36].

The involved metabolic pathways showed different importance in IVF/ICSI outcomes in different groups. In the tubal group, glycolysis, along with pyruvate metabolism, was a determinant in IVF/ICSI outcomes. Generally, the energy supporting the oocyte to undergo fertilization, cleavage, and proliferation was provided via glycolysis, in which glucose was converted to pyruvate and entered a tricarboxylic acid (TCA) cycle [37]. Consumption of lactate and amino acids was helpful in the process. In the dysspermia group, similar to what we found in our previous study [32], amino acids such as leucine and isoleucine were negatively correlated with IVF/ICSI outcomes. It was consistent with a study in which repeated IVF failure found an elevation of 11 amino acids in follicular fluids [8]. The predominant role of amino acids highlighted aminoacyl-tRNA biosynthesis, in which amino acids were delivered to the ribosome and incorporated into the polypeptide chain for protein synthesis. Investigated in different species, the deficiency of amino acids has been related to chromosome aneuploidy [38], DNA damage [39], etc., and ultimately revealed its relationship with oocyte quality. The adequate consumption of amino acids in protein synthesis helps oocytes prepare for their high-quality status to get ready for following tough fertilization and development processes because of the sperm defects. On the other hand, the energy production related to arginine and proline metabolism, as well as alanine, aspartate, and glutamate metabolism, was also provoked to provide support.

The most unfortunate thing about our study is that we did not have the opportunity to trace further the molecular biology of sperm other than the ordinary clinical sperm parameters. Combination analysis of paternal DNA fragmentation, chromosomal aneuploidies, and/or epigenetic mutations would be of expectation in future work. On the other hand, the relatively small number of the dysspermia sample group was the major limitation. Moreover, the logistic regression model in the current study pointed out the possibility of using follicular metabolites and couples' physical parameters to predict IVF/ICSI outcomes objectively. More sample cohorts are expected to improve and verify the model for clinical application.

## 5. Conclusion

The present study explored the relationship among couples' clinical factors, follicular metabolites, and IVF/ICSI stepwise outcomes. Our findings indicated that in the case of fallopian tubal blockage as a cause of female infertility, the factors of both males (sperm volume and viability, etc.) and females (follicular alanine, glutamine, and cytoplasmic status, etc.) need to be evaluated, while in the case of male infertility caused by sperm problems, oocyte quality in terms of the follicular metabolites index (especially glycoprotein, lipids, and acetic acid) as a compensatory role should be considered. In the treatment of infertility, synergistic effects from both sexes cannot be ignored. In addition, ovary stimulation protocols should be adjusted with the concern of both the disease and female age to achieve proper ORN. Despite the limited sample size, the study provided us with a feasible option for pre-IVF/ICSI evaluation from a molecular point of view and constitutes a framework for future intervention improvement by considering both female and male factors.

## Figures and Tables

**Table 1:** Representative demographic information by group.

Clinical characteristic	Tubal group (*n* = 75)	Dysspermia group (*n* = 42)	Corrected *p* value (95% CI)
*Females*			
Age	32.0 ± 5.1	31.1 ± 4.9	NS
BMI (kg/m^2^)	22.2 ± 3.3	21.6 ± 2.9	NS
Basal FSH (mIU/ml)	6.2 ± 1.7	6.9 ± 2.4	NS
Basal LH (mIU/ml)	4.1 ± 2.0	4.4 ± 2.2	NS
Estradiol, (E2, pg/ml)	40.6 ± 15.9	48.7 ± 22.1	NS
BP sys. (mmHg)	101.6 ± 3.8	101.9 ± 3.3	NS
BP dia. (mmHg)	65.5 ± 6.3	64.4 ± 5.5	NS
Pulse rate (P., min^−1^)	66.1 ± 7.2	66.3 ± 7.3	NS
Respiration rate (R., min^−1^)	20.2 ± 1.1	20.3 ± 1.1	NS
DBIL (umol/L)	3.6 ± 2.1	3.5 ± 1.9	NS
BUN (mmol/L)	4.2 ± 1.1	4.3 ± 1.1	NS
Fasting blood glucose (mmol/L)	5.0 ± 1.3	4.8 ± 1.2	NS
Creatinine, (Cr, *μ*mol/L)	49.8 ± 8.1	51.8 ± 10.3	NS
SFN	13 ± 6	12 ± 6	NS
ORN	12 ± 7	12 ± 6	NS

*Males*			
Age	33.5 ± 8.0	33.6 ± 5.4	NS
BMI (kg/m^2^)	22.7 ± 6.4	23.4 ± 6.4	NS
BP sys. (mmHg)	108.3 ± 23.6	106.3 ± 24.7	NS
BP dia. (mmHg)	68.0 ± 16.1	66.1 ± 16.2	NS
Sperm density (×10^6^/mg)	88.6 ± 52.1	44.0 ± 48.2	<0.01
Sperm motility A (%)	22.9 ± 8.6	12.7 ± 10.3	<0.01
Sperm motility B (%)	37.3 ± 8.5	23.7 ± 15.2	<0.01
Level a sperm (%)^1^	13.6 ± 9.0	3.3 ± 4.0	<0.01
Level b sperm (%)^2^	21.0 ± 11.1	7.5 ± 7.5	<0.01
Total motility (PR + NP, %)	36.4 ± 11.5	14.9 ± 9.3	<0.01

FSH, follicle-stimulating hormone; LH, luteinizing hormone; BP sys., systolic blood pressure; BP dia., diastolic blood pressure; DBIL, direct bilirubin; BUN, blood urea nitrogen; PR, progressive; NP, nonprogressive; SFN, small follicle number in ovaries; ORN, oocyte retrieved number. Level a sperm: fast-moving sperms; Level b sperm: slowly moving sperms.

**Table 2:** Logistic regression model for tubal group.

	Coefficient details					Model parameter				
	B	Sig.	Exp (B)	95% CI for exp (B) lower bound	95% CI for exp (B) upper bound	Model -2 log-likelihood	Overall predicted percentage	ROC area	95% CI lower Bound	95% CI upper Bound
*IVF fertilization rate for tubal group*										
Alanine	1.92*E* + 02	0.013	1.83*E* + 83	4.76*E* + 17	7.01*E* + 148	60.60^a^	83.82	0.85	0.76	0.95
E2 pg ml^−1^	−5.37*E* − 02	0.016	9.48*E* − 01	9.07*E* − 01	9.90*E* − 01					
Cytoplasmic states (1. granular; 2. homogeneous)	2.64*E* + 00	0.001	1.41*E* + 01	2.94*E* + 00	6.72*E* + 01					
Sperm viability	1.16*E* − 01	0.002	1.12*E* + 00	1.04*E* + 00	1.21*E* + 00					

*IVF top-quality embryos rate for tubal group*										
Glutamine	2.87*E* + 02	0.012	2.72*E* + 124	6.56*E* + 27	1.13*E* + 221	69.92^a^	80.88	0.83	0.73	0.93
Sperm volume (mL)	1.22*E* + 00	0.003	3.38*E* + 00	1.52*E* + 00	7.52*E* + 00					
Sperm viability	6.98*E* − 02	0.022	1.07*E* + 00	1.01*E* + 00	1.14*E* + 00					
Oocyte radial crown arrangement (1. compact; 2. slightly dilated; 3. radial)	1.62*E* + 00	0.005	5.03*E* + 00	1.62*E* + 00	1.56*E* + 01					

^a^Estimation terminated at iteration number 6 because parameter estimates changed by less than 0.001.

**Table 3:** Logistic regression models for the dysspermia group.

	Coefficient details					Model parameter				
	B	Sig.	Exp (B)	95% CI for exp (B) lower Bound	95% CI for exp (B) upper Bound	Model -2 log-likelihood	Overall predicted percentage	ROC area	95% CI lower Bound	95% CI upper Bound
*IVF fertilization rate for dysspermia group*										
Glycoprotein	−3.43*E* + 02	0.035	8.57*E* − 150	2.27*E* − 288	3.23*E* − 11	17.96^a^	75.00	0.84	0.67	1.00

*IVF top-quality embryos rate for dysspermia group*										
Lipid	6.00*E* + 02	0.028	5.02*E* + 260	4.37*E* + 28		10.97^b^	90.00	0.96	0.87	1.00
ALT	−4.69*E* − 01	0.040	6.26*E* − 01	4.00*E* − 01	9.80*E* − 01					

*ICSI top-quality embryos rate for dysspermia group*										
Acetate	5.73*E* + 02	0.025	8.06*E* + 248	3.80*E* + 31		29.09^c^	76.92	0.81	0.64	0.98

^a^Estimation terminated at iteration number 6 because parameter estimates changed by less than 0.001. ^b^Estimation terminated at iteration number 9 because parameter estimates changed by less than 0.001. ^c^Estimation terminated at iteration number 5 because parameter estimates changed by less than 0.001.

## Data Availability

The data underlying this article are available upon reasonable request to the corresponding author.

## Abbreviations

Ala: Alanine
ALT: Alanine aminotransferase
Arg: Arginine
ART: Assisted reproductive technology
AUROC: Area under ROC
Ave: Average
BMI: Body mass index
BP sys.: Systolic blood pressure
BP dia.: Diastolic blood pressure
CI: Confidence interval
CPMG: Carr-Purcell-Meiboom-Gill
Cr: Creatinine
CRE: Creatine
Cyto. Stat.: Cytoplasmic states
E2: Estradiol
ECM: Embryo culture medium
FR: Fertilization rate
FSH: Follicle-stimulating hormone
Gln: Glutamine
Glu: Glucose
GPro: Glycoprotein
HCG: Human chorionic gonadotropin
His: Histidine
IRB: Institutional review boards
ICSI: Intracytoplasmic sperm injection
Ile: Isoleucine
IVF: In vitro fertilization
Leu: Leucine
LH: Luteinizing hormone
Lys: Lysine
PR: Progressive
Pro: Proline
NP: Nonprogressive
OCRA: Oocyte radial crown arrangement (1. compact; 2. slightly dilated; 3. radial)
ORN: Oocyte retrieved number
ROC: Receiver operating characteristic
STD: Standard deviation
SFN: Small follicle number
SFN: Small follicle number
Sperm Den.: Sperm density
Sperm malFR: Sperm malformation rate
Sperm NM: Sperm normal morphology (%)
Sperm Via.: Sperm viability
Sperm Vol.: Sperm volume
TQER: Top-quality embryos rate
U. Cr.: Urinary creatinine
Val: Valine
2PN: Two pronuclei
2PNFR: 2PN fertilization rate
3HBA: 3-Hydroxybutyric acid.

## Supplementary Materials

Supplementary Materials Figure S1: summary of factors impacting stepwise IVF/ICSI outcomes by group. PR, progressive; NP, nonprogressive; Sperm NM, sperm normal morphology (%); Sperm Via., sperm viability; Sperm Vol., sperm volume; Sperm Den., sperm density; Sperm malFR, Sperm malformation rate; BP sys., systolic blood pressure; BP dia., diastolic blood pressure; FSH, follicle-stimulating hormone; E2, estradiol; Cyto. Stat., cytoplasmic states; U. Cr., urinary creatinine; Gln, glutamine; Oocyte arr., oocyte radial crown arrangement (1.compact; 2. slightly dilated; 3. radial); SFN, small follicle number in ovaries; ORN, oocyte retrieved number; ALT, alanine aminotransferase; Ala, alanine; His, Histidine; Glu, glucose; GPro, glycoprotein; Lys, lysine; Ile, Isoleucine; Leu, leucine; Cr, creatinine; CRE, creatine; 3HBA, 3-Hydroxybutyric acid; Val, valine; Gln, glutamine; Pro, proline. Table S1: embryo quality evaluation strategy on day 3. Table S2: integrated 67 metabolite regions. Table S3: stepwise ART outcomes by group. Table S4: correlation coefficients between clinical parameters and IVF/ICSI outcomes in the tubal group. Table S5: correlation coefficients between follicular metabolites and IVF/ICSI outcomes in the tubal group. Table S6: correlation coefficients between clinical parameters and ART outcomes in dysspermia group. Table S7: correlation coefficients between follicular metabolites and ART outcomes in dysspermia group. Table S8: summary of metabolic pathway enrichment analysis.
