# Supplementary material for: Follicular Metabolites-Assisted Clinical Evaluation of IVF/ICSI Outcomes
Source: Evid Based Complement Alternat Med. 2021 May 26;2021:9999659. doi: 10.1155/2021/9999659 (PMC8189786; doi:10.1155/2021/9999659)
Supplement: Supplementary Materials — Figure S1: summary of factors impacting stepwise IVF/ICSI outcomes by group. PR, progressive; NP, nonprogressive; Sperm NM, sperm normal morphology (%); Sperm Via., sperm viability; Sperm Vol., sperm volume; Sperm Den., sperm density; Sperm malFR, Sperm malformation rate; BP sys., systolic blood pressure; BP dia., diastolic blood pressure; FSH, follicle-stimulating hormone; E2, estradiol; Cyto. Stat., cytoplasmic states; U. Cr., urinary creatinine; Gln, glutamine; Oocyte arr., oocyte radial crown arrangement (1.compact; 2. slightly dilated; 3. radial); SFN, small follicle number in ovaries; ORN, oocyte retrieved number; ALT, alanine aminotransferase; Ala, alanine; His, Histidine; Glu, glucose; GPro, glycoprotein; Lys, lysine; Ile, Isoleucine; Leu, leucine; Cr, creatinine; CRE, creatine; 3HBA, 3-Hydroxybutyric acid; Val, valine; Gln, glutamine; Pro, proline. Table S1: embryo quality evaluation strategy on day 3. Table S2: integrated 67 metabolite regions. Table S3: stepwise ART outcomes by group. Table S4: correlation coefficients between clinical parameters and IVF/ICSI outcomes in the tubal group. Table S5: correlation coefficients between follicular metabolites and IVF/ICSI outcomes in the tubal group. Table S6: correlation coefficients between clinical parameters and ART outcomes in dysspermia group. Table S7: correlation coefficients between follicular metabolites and ART outcomes in dysspermia group. Table S8: summary of metabolic pathway enrichment analysis. [file 9999659.f1.docx]

**Supplementary Information**


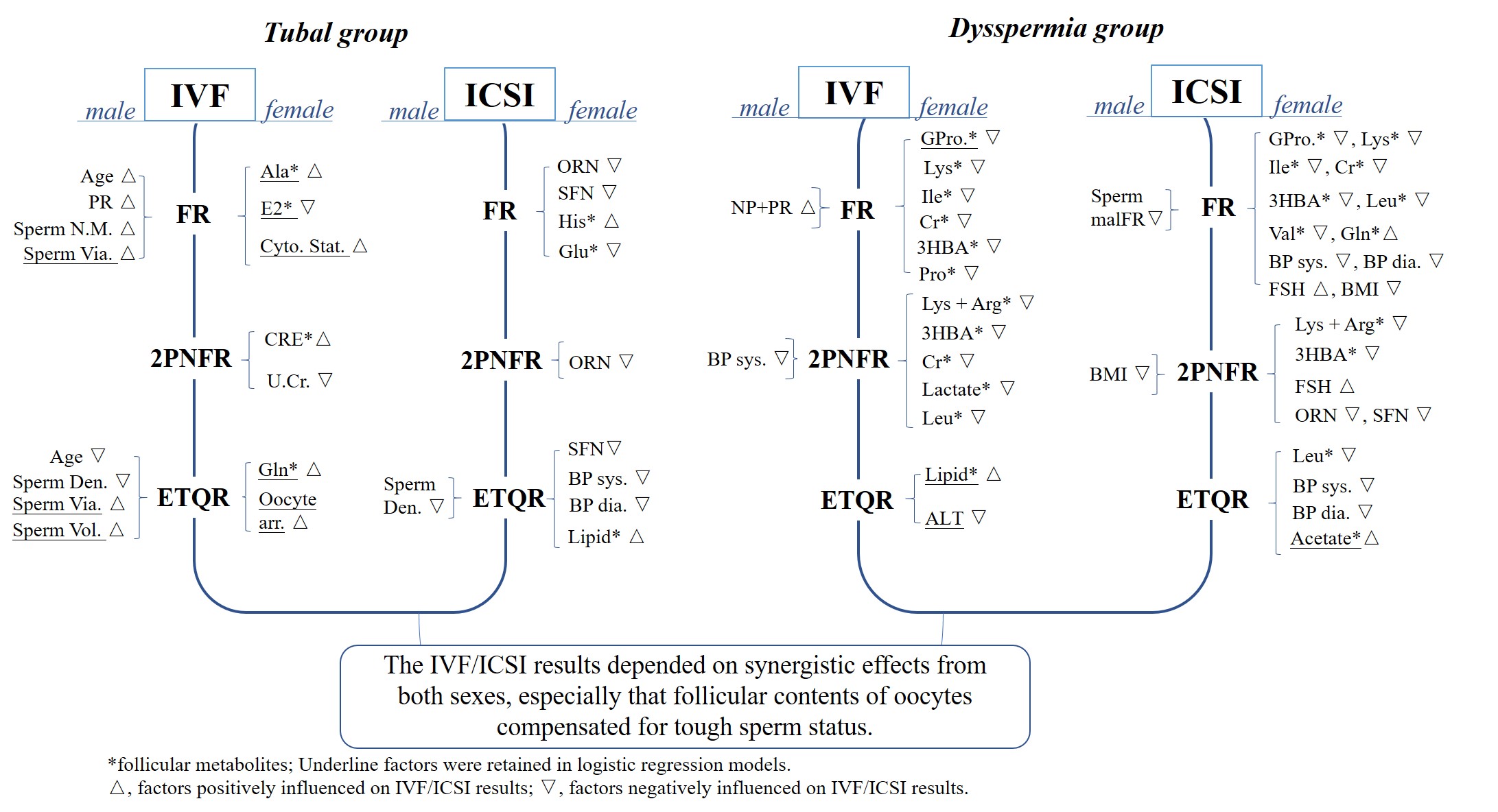


Fig. S1. Summary of factors impacting on stepwise IVF/ICSI outcomes by group. PR, progressive; NP, non-progressive; Sperm NM, sperm normal morphology (%); Sperm Via., sperm viability; Sperm Vol., sperm volume; Sperm Den., sperm density; Sperm malFR, Sperm malformation rate; BP sys., systolic blood pressure; BP dia., diastolic blood pressure; FSH, follicle stimulating hormone; E2, estradiol; Cyto. Stat., cytoplasmic states; U. Cr., urinary creatinine; Gln, glutamine; Oocyte arr., oocyte radial crown arrangement (1.compact; 2. slightly dilated; 3. radial); SFN, small follicle number in ovaries; ORN, oocyte retrieved number; ALT, alanine aminotransferase ; Ala, alanine; His, Histidine; Glu, glucose; GPro, glycoprotein; Lys, lysine; Ile, Isoleucine; Leu, leucine; Cr, creatinine; CRE, creatine; GLU, glucose; 3HBA, 3-Hydroxybutyric acid; Val, valine; Gln, glutamine; Pro, proline.

Table S1. Embryo quality evaluation strategy on day 3.

| Grade | Size & shape | Fragments in percentage (%) |
| --- | --- | --- |
| I | Equivalent | None |
| II | Slightly different | Less than 20% |
| III | Significantly different | Between 20% and 50% |

Table S2, Integrated 67 metabolite regions.

| **No.** | **Metabolite** | **Chemical shift (ppm)** | **Multiplicity** | **No.** | **Metabolite** | **Chemical shift (ppm)** | **Multiplicity** |
| --- | --- | --- | --- | --- | --- | --- | --- |
| **Pk 1** | formate | 8.45 | s | **Pk 35** | creatine | 3.01 | s |
| **Pk 2** | histidine | 7.73 | s | **Pk 36** | lysine | 3.00 | t |
| **Pk 3** | tyrosine | 7.17 | m | **Pk 37** | aspartate | 2.86 | dd |
| **Pk 4** | histidine | 7.03 | s | **Pk 38** | trimethylamine | 2.84 | s |
| **Pk 5** | tyrosine | 6.88 | m | **Pk 39** | aspartate | 2.82 | dd |
| **Pk 6** | urea | 5.76 | br. s. | **Pk 40** | dimethylamine | 2.73 | s |
| **Pk 7** | α-glucose | 5.23 | d | **Pk 41** | citrate | 2.52 | d |
| **Pk 8** | β-glucose | 4.64 | d | **Pk 42** | glutamine | 2.43 | m |
| **Pk 9** | creatinine | 4.04 | s | **Pk 43** | glutamate | 2.38 | m |
| **Pk 10** | creatine | 3.92 | s | **Pk 44** | pyruvate | 3.36 | s |
| **Pk 11** | β-glucose | 3.90 | dd | **Pk 45** | 3-hydroxybutyrate | 2.33 | m |
| **Pk 12** | β-glucose | 3.88 | dd | **Pk 46** | acetoacetate | 2.22 | s |
| **Pk 13** | glucose | 3.85 | dd | **Pk 47** | glutamine | 2.12 | m |
| **Pk 14** | α-glucose | 3.83 | ddd | **Pk 48** | proline | 2.05 | m |
| **Pk 15** | glucose | 3.70 | t | **Pk 49** | glycoprotein | 2.03 | s |
| **Pk 16** | glucose | 3.69 |  | **Pk 50** | proline | 1.98 | m |
| **Pk 17** | glycerol + lipid | 3.65 | m | **Pk 51** | acetate | 1.91 | s |
| **Pk 18** | glycine* | 3.54 | s | **Pk 52** | lysine + arginine* | 1.87 | m |
| **Pk 19** | α-glucose | 3.52 | dd | **Pk 53** | lysine + arginine* | 1.70 | m |
| **Pk 20** | glucose | 3.50 | s | **Pk 54** | alanine | 1.47 | d |
| **Pk 21** | β-glucose | 3.48 | t | **Pk 55** | lactate | 1.32 | d |
| **Pk 22** | glucose | 3.47 | ddd | **Pk 56** | lipids | 1.29 | 一 |
| **Pk 23** | glucose | 3.44 |  | **Pk 57** | lipids | 1.36 | 一 |
| **Pk 24** | glucose | 3.43 | t | **Pk 58** | 3-hydroxybutyrate | 1.19 | d |
| **Pk 25** | glucose | 3.39 | t | **Pk 59** | unknown1 | 1.06 | d |
| **Pk 26** | glucose | 3.37 | t | **Pk 60** | valine | 1.03 | d |
| **Pk 27** | TMAO | 3.35 | s | **Pk 61** | isoleucine | 1.00 | d |
| **Pk 28** | myo-inositol (tentatively) | 3.29 | t | **Pk 62** | valine | 0.97 | d |
| **Pk 29** | β-glucose | 3.26 | dd | **Pk 63** | leucine | 0.95 | d |
| **Pk 30** | β-glucose | 3.24 | dd | **Pk 64** | leucine | 0.94 | d |
| **Pk 31** | choline | 3.22 | s | **Pk 65** | isoleucine+leucine* | 0.93 | t |
| **Pk 32** | phosphocholine | 3.19 | s | **Pk 66** | isoleucine | 0.91 | d |
| **Pk 33** | phenylalanine | 3.12 | dd | **Pk 67** | cholesterol | 0.82 | m |
| **Pk 34** | creatinine | 3.03 | s |  |  |  |  |

Table S3. Stepwise ART outcomes by group.

|  | Tubal group | Dysspermia group |
| --- | --- | --- |
| IVF | | |
| IVF number | 68 | 20 |
| IVF FR (%) | 62.4 ± 26.9 | 59.3 ± 26.1 |
| IVF 2PNR (%) | 76.7 ± 28.0 | 85.5 ± 16.6 |
| IVF ETQR (%) | 67.6 ± 32.2 | 60.0 ± 36.5 |
| ICSI | | |
| ICSI number | 21 | 26 |
| ICSI FR (%) | 82.7 ± 16.0 | 81.9 ± 14.2 |
| ICSI 2PNR (%) | 87.3 ± 22.3 | 90.9 ± 15.1 |
| ICSI ETQR (%) | 50.2 ± 35.1 | 53.2 ± 28.0 |
| Overall ART (combing IVF and ICSI) | | |
| Overall number | 75 | 42 |
| Overall FR (%) | 69.2 ± 18.9 | 72.8 ± 21.7 |
| Overall 2PNR (%) | 82.5 ± 20.9 | 89.0 ±14.7 |
| Overall ETQR (%) | 64.9 ± 30.3 | 56.1 ± 31.3 |

Table S4. Correlation coefficients between clinical parameters and IVF/ICSI outcomes in tubal group.

| Clinical parameters | Overall ART result | IVF | ICSI |
| --- | --- | --- | --- |
| Fertilizaiton rate (FR) | | | |
| Age^a^ | 0.33** | 0.29* | -0.11 |
| Sperm normal morphology^a^ (%) | 0.20 | 0.27* | -0.04 |
| PR^a^ (%) | 0.17 | 0.26* | -0.20 |
| Oocyte retrieved number^b^ | -0.22 | -0.13 | -0.52* |
| Small follicles in ovaries^b^ | -0.12 | 0.03 | -0.47* |
| 2PN fertilization rate (2PNR) | | | |
| Urinary creatinine^b^ (umol/L) | -0.38** | -0.48** | -0.01 |
| Oocyte retrieved number^b^ | -0.24* | -0.06 | -0.63** |
| Embryos' top-quality rate (ETQR) | | | |
| Sperm density^a^ (×10^6/mg) | -0.36** | -0.35** | -0.48* |
| Age^a^ | -0.32** | -0.28* | 0.09 |
| Small follicles in ovaries^b^ | -0.09 | 0.059 | -0.65** |
| BP sys. ^b^ (mmHg) | 0.02 | 0.14 | -0.51* |
| BP dia. ^b^ (mmHg) | -0.17 | -0.11 | -0.58** |
| ^a^ Males' data; ^b^ Females' data. |  |  |  |
| ** p-value < 0.01; *p-value < 0.03 |  |  |  |

Table S5. Correlation coefficients between follicular metabolites and IVF/ICSI outcomes in tubal group.

| Follicular metabolites | Overall ART result | IVF | ICSI |
| --- | --- | --- | --- |
| Fertilizaiton rate (FR) | | | |
| Lactate | 0.31** | 0.20 | 0.20 |
| Alanine | 0.30** | 0.24* | -0.05 |
| Histidine | 0.16 | 0.08 | 0.56** |
| Glucose | 0.00 | 0.05 | -0.51* |
| 2PN fertilization rate (2PNR) | | | |
| β-glucose | -0.29* | -0.22 | 0.08 |
| Creatine | 0.28* | 0.28* | 0.21 |
| Embryos' top-quality rate (ETQR) | | | |
| Lipid | 0.07 | 0.01 | 0.52* |
| ** p-value < 0.01; *p-value < 0.05 | |  |  |

Table S6. Correlation coefficients between clinical parameters and ART outcomes in dysspermia group.

| Clinical parameters | Overall ART result | IVF | ICSI |
| --- | --- | --- | --- |
| Fertilizaiton rate (FR) | | | |
| NP+PR^a^ (%) | -0.06 | 0.59** | -0.19 |
| Malformation rate^a^ (%) | -0.16 | 0.05 | -0.47* |
| FSH^b^（mIU/ml) | 0.42** | 0.37 | 0.49* |
| BMI^b^ | -0.06 | -0.02 | -0.48* |
| BP sys.^b^ (mmHg) | -0.35* | -0.08 | -0.42* |
| BP dia.^b^ (mmHg) | -0.26 | -0.04 | -0.53** |
| 2PN fertilization rate (2PNR) | | | |
| BMI^a^ | -0.35* | -0.26 | -0.55** |
| BP sys.^a^ (mmHg) | -0.20 | -0.51* | 0.15 |
| FSH^b^（mIU/ml) | 0.01 | -0.24 | 0.57** |
| Occyte retrieved number^b^ | -0.26 | -0.07 | -0.45* |
| Small follicles in ovaries^b^ | -0.35* | -0.26 | -0.42* |
| Embryos' top-quality rate (ETQR) | | | |
| BP sys.^b^ (mmHg) | -0.18 | 0.02 | -0.48* |
| BP dia.^b^ (mmHg) | -0.3 | -0.08 | -0.55** |
| ^a^ Males' data; ^b^ Females' data. | ** p-value < 0.01; *p-value < 0.03 | | |

Table S7. Correlation coefficients between follicular metabolites and ART outcomes in dysspermia group.

| Follicular metabolites | Overall ART result | IVF | ICSI |
| --- | --- | --- | --- |
| Fertilizaiton rate (FR) | | | |
| Glycoprotein | -0.65** | -0.70** | -0.55** |
| Lysine | -0.60** | -0.68** | -0.39* |
| Isoleucine | -0.52** | -0.47* | -0.58** |
| Creatinine | -0.64** | -0.52* | -0.58** |
| 3-Hydroxybutyric acid | -0.65** | -0.52* | -0.55** |
| Proline | -0.52** | -0.69** | -0.30 |
| Leucine | -0.49** | -0.42 | -0.63** |
| Valine | -0.48** | -0.37 | -0.51** |
| Glutamine | 0.28 | -0.17 | 0.50** |
| 2PN fertilization rate (2PNR) | | | |
| Lysine+arginine | -0.50** | -0.79** | -0.40* |
| 3-Hydroxybutyric acid | -0.46** | -0.60** | -0.47* |
| Creatinine | -0.54** | -0.70** | -0.36 |
| Lactate | -0.39* | -0.68** | -0.08 |
| Leucine | -0.35* | -0.62** | -0.24 |
| Embryos' top-quality rate (ETQR) | | | |
| Leucine | -0.05 | -0.02 | -0.46* |
| ** p-value < 0.01; *p-value < 0.03 | |  |  |

Table S8. Summary of metabolic pathway enrichment analysis.

| Metabolic pathway | Total | Expected | Hits | p-value | FDR | Impact |
| --- | --- | --- | --- | --- | --- | --- |
| Aminoacyl-tRNA biosynthesis | 75 | 0.4674 | 9 | 7.34E-11 | 5.88E-09 | 5.63E-02 |
| Arginine and proline metabolism | 77 | 0.4799 | 5 | 6.87E-05 | 2.75E-03 | 2.67E-01 |
| Valine, leucine and isoleucine biosynthesis | 27 | 0.1683 | 3 | 5.24E-04 | 1.40E-02 | 3.98E-02 |
| Valine, leucine and isoleucine degradation | 40 | 0.2493 | 3 | 1.69E-03 | 3.37E-02 | 2.23E-02 |
| Alanine, aspartate and glutamate metabolism | 24 | 0.1496 | 2 | 9.25E-03 | 1.48E-01 | 2.64E-01 |
| Glycolysis or Gluconeogenesis | 31 | 0.1932 | 2 | 1.52E-02 | 2.03E-01 | 0 |
| Propanoate metabolism | 35 | 0.2181 | 2 | 1.92E-02 | 2.19E-01 | 0 |
| Nitrogen metabolism | 39 | 0.2430 | 2 | 2.35E-02 | 2.35E-01 | 0 |
| Synthesis and degradation of ketone bodies | 6 | 0.0374 | 1 | 3.69E-02 | 3.28E-01 | 0 |
| D-Arginine and D-ornithine metabolism | 8 | 0.0499 | 1 | 4.89E-02 | 3.91E-01 | 0 |
